# Closing 2019 with a golden key

**DOI:** 10.20945/2359-3997000000182

**Published:** 2019-11-01

**Authors:** Marcello D. Bronstein

**Affiliations:** 1 Hospital das Clínicas Faculdade de Medicina Universidade de São Paulo São Paulo SP Brasil Editor-chefe da Archives of Endocrinology and Metabolism, chefe da Unidade de Neuroendocrinologia, Serviço de Endocrinologia e Metabologia, Hospital das Clínicas, Faculdade de Medicina da Universidade de São Paulo (HCFMUSP), São Paulo, SP, Brasil

When I invited Drs Cesar and Margaret Boguszewski ( 1 ) as guest editors of the AE&M special issue on “Growth, Growth hormone and related disorders” I was absolutely confident about the outcome. In fact, due to their international expertise in the field, they gathered an outstanding team of experts who contributed with excellent articles. I heartily thank the invited editors as well as all colleagues who contributed with their precious time and knowledge to enrich this last 2019 issue of our Journal.
